# The texture-taste connection: Multimodal sensory neurons in fly larvae

**DOI:** 10.1371/journal.pbio.3003000

**Published:** 2025-01-31

**Authors:** Katrin Vogt

**Affiliations:** 1 Department of Biology, University of Konstanz, Konstanz, Germany; 2 Centre for the Advanced Study of Collective Behaviour, University of Konstanz, Konstanz, Germany

## Abstract

Eating is a multisensory experience: food’s smell, look, and texture are as important as taste. This Primer explores a new study in PLOS Biology showing that fly larvae respond to food texture and integrate information from different modalities within a single gustatory neuron.

## Teaser

Eating is a multisensory experience: food’s smell, look, and texture are as important as taste. A new study in *PLOS Biology* shows that fly larvae respond to food texture and integrate information from different modalities within a single gustatory neuron.

We enjoy biting into a fresh and crispy apple, indulge in a creamy chocolate mousse, and get addicted to crunchy potato chips. What makes these foods so appealing? Of course, they taste good; the apple and chocolate mousse are sweet, and the potato chips are salty and spicy, but their texture adds a lot to the eating experience. Imagine creamy potato chips or slimy apples—that doesn’t sound right or delicious.

**Fig 1:**
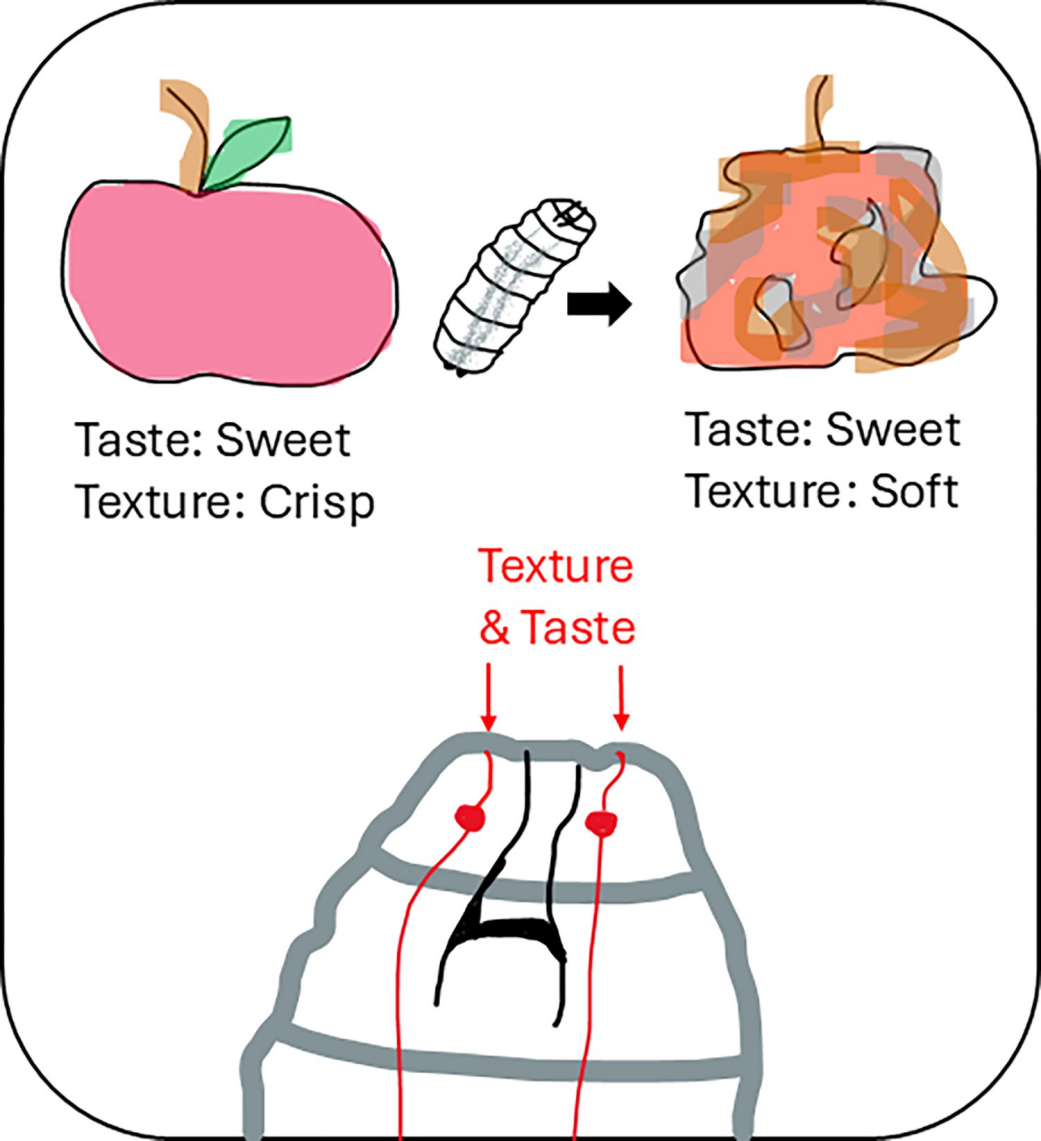
The taste-texture sensation is encoded in single sensory neurons in the fly larva. Fly larvae evaluate food texture and choose soft rotten fruits over harder ripe ones, even though both contain sweet fructose. Sweet taste and soft texture information activate a single bilaterally expressing sensory neuron type in the larval head, projecting to the larval brain.

We often do not realize that in addition to taste, the look, temperature, texture, and even sound of food produced when we eat it are important for a satisfying and palatable dining experience. Food texture is essential to evaluate the freshness of the food—a mushy apple indicates that it is old and potentially rotten [1] (Fig 1). While many neuroethological studies investigate visual, taste, and olfactory processing, it is less well understood how food texture is sensed. We also do not know yet where all the sensory information channels are integrated within the nervous system to allow for a multisensory eating experience.

In their new *PLOS Biology* study, Komarov and colleagues investigate the role and mechanism of food hardness sensing in the larva of *Drosophila melanogaster*, the common fruit fly [2]. Fruit fly larvae are the feeding stage of the fly. They prefer rotten fruits and require a carbohydrate- and protein-rich diet to achieve a 200-fold increase in body mass before pupation. In line with previous studies [3–5], Komarov and colleagues show that fly larvae have a soft spot, they prefer and ingest specific agarose substrate concentrations (0.5% to 1.5%), but avoid and do not ingest softer and harder substrates. Fresh fruits fall into the hard substrate category, whereas fruits that decompose over several days fall into the accordingly ecologically relevant soft spot (Fig 1). The texture of the substrate, which is usually their food, is so important to them, that it can even be used as a reinforcer during associative conditioning [5]. After learning, the larva will navigate toward the odor previously presented with a soft spot substrate. Larvae not only eat the soft substrate but also need to be able to dig into the food to protect themselves and to reach deeper, better fruit parts. As this is only possible if the food is soft enough, they need to be able to accurately evaluate the substrate hardness [4].

So, how do they do that? Fruit flies are an established model organism in neuroscience with a rich genetic toolkit and a complete neuronal wiring diagram, thus they are perfectly suited to unravel the neural mechanisms and circuits underlying food texture sensing. In flies, mechanosensory neurons inhibit sweet sensory neurons to prevent eating when the substrate is too hard [6]. In the larva, similarly, a mechanoreceptor called “painless,” is involved in food substrate sensing and ingestion; however, the neural mechanism underlying taste-texture integration is different [2]. The Sprecher lab previously found that gustatory sensory neurons in the larvae are often multisensory and express multiple different receptors, thus they hypothesized that they could also respond to texture [7]. In the new study, using a genetic trick allowed for the manipulation of all gustatory sensory neurons in the larva and to test for their role in texture sensing. They searched for proteins that are expressed in gustatory sensory neurons without overlap in other cell types. Making use of the prominent GAL4-UAS binary expression system, combining two of such protein expression patterns created an intersectional gustatory sensory neuron-specific Split-GAL4 driver line. As expected, silencing all gustatory sensories using this new line made larvae taste-blind to sweet and bitter. However, it also revealed that they could not differentiate between soft and hard substrates. Without the mechanosensory receptor in these neurons, larvae even ingested very hard substrates, which everybody thought they were not capable of eating.

Functional imaging further revealed that indeed single gustatory sensory neurons are activated by changes in water pressure, in addition to sweet or CO_2_ stimuli. These findings add to the growing evidence that most sensory neurons are not specifically tuned to one receptor type, but that they already integrate information from different receptors. The roundworm *Caenorhabditis elegans* was the odd one for a long time, having few sensory neurons, each with multiple receptors. However, a fly study recently showed that olfactory receptor neurons contain multiple other non-olfactory chemosensors [8] with unknown functions. Also in mosquitoes, many sensory neurons express multiple different chemosensors, allowing for complex coding and robust human feature extraction [9]. Komarov and colleagues now suggest that chemosensors not only coexpress with other types of chemosensors but also with sensors from other modalities, such as mechanosensors [2] (Fig 1). Silencing single mechanoreceptors in a single sensory neuron however cannot reproduce the phenotype found when silencing all gustatory neurons—suggesting redundancy across neurons. In the case of taste-texture sensing, the discovered receptor combination might have been evolutionarily learned by the nervous system, as larval food usually comes together with a sweet taste and CO_2_ produced from soft rotting fruits. Thus, to understand the function of other unknown receptor combinations, we should keep in mind the ecology of the animals to understand what sensory contexts they are exposed to and how these contexts are relevant to them. Additional senses, such as temperature might also play an important role in food consumption and could be integrated in early processing stages.

As mentioned before, food texture sensing is crucial for the human eating experience. Harder, crunchy foods take longer to eat, make more noise, and thus seem to provide a more satisfactory eating experience. Such information can not only be useful to fight obesity but also to enhance food consumption in poor third-world countries. With the growing and aging human population, dependence on artificial food with specific textures made in the lab or by 3D printers might increase [10]. A better understanding of the important role of food texture and how it is integrated with other sensory modalities will help us create novel satisfying and also palatable food.
